# The Association between Meditation Practice and Job Performance: A Cross-Sectional Study

**DOI:** 10.1371/journal.pone.0128287

**Published:** 2015-05-29

**Authors:** Koichiro Shiba, Masahiro Nishimoto, Minami Sugimoto, Yoshiki Ishikawa

**Affiliations:** 1 Department of Health and Social Behavior/Health Education and Health Sociology, School of Public Health, The University of Tokyo, Tokyo, Japan; 2 Campus for H Inc., Tokyo, Japan; 3 Department of Social and Preventive Epidemiology, School of Public Health, The University of Tokyo, Tokyo, Japan; University of Utah, UNITED STATES

## Abstract

Many previous studies have shown that meditation practice has a positive impact on cognitive and non-cognitive functioning, which are related to job performance. Thus, the aims of this study were to (1) estimate the prevalence of meditation practice, (2) identify the characteristics of individuals who practice meditation, and (3) examine the association between meditation practice and job performance. Two population-based, cross-sectional surveys were conducted. In study 1, we examined the prevalence of meditation practice and the characteristics of the persons practicing meditation; in Study 2, we examined the association between meditation practice and job performance. The outcome variables included work engagement, subjective job performance, and job satisfaction. The Utrecht Work Engagement Scale was used to assess work engagement, the World Health Organization Health and Work Performance Questionnaire (HPQ) was used to measure subjective job performance, and a scale developed by the Japanese government was used to assess job satisfaction. Hierarchical multiple regression analyses were used in Study 2. Demographic characteristics and behavioral risk factors were included as covariates in the analyses. The results of Study 1 indicated that 3.9% of persons surveyed (*n* = 30,665) practiced meditation; these individuals were younger and had a higher education, higher household income, higher stress level, and lower body mass index than those who did not practice meditation. The results of Study 2 (*n* = 1,470) indicated that meditation practice was significantly predictive of work engagement (*β* = 0.112, *p* < .001), subjective job performance (*β* = 0.116, *p* < .001), and job satisfaction (*β* = 0.079, *p* = .002), even after adjusting for covariates (*β* = 0.083, *p* < .001; *β* = 0.104, *p* < .001; *β* = 0.060, *p* = .015, respectively). The results indicate that meditation practice may positively influence job performance, including job satisfaction, subjective job performance, and work engagement.

## Introduction

The improvement of employee job performance has been an issue of interest in the fields of management, occupational health, and organizational psychology [1]. It is well documented that cognitive intelligence is positively associated with job performance [2]. Furthermore, researchers have also focused on non-cognitive factors that are positively associated with job performance. For instance, higher emotional intelligence has been linked to better job performance [3, 4], as has motivation, which can be fostered by vocational interest [5]. While it is known that these factors are positively associated with job performance, our understanding of a practical means of enhancing job performance remains insufficient.

One promising intervention that may improve individual job performance is meditation practice. Meditation training has been used as a tool in psychotherapy [6], and has recently been introduced to the field of business. Indeed, meditation practice may have a positive impact on concentration, memory, creativity, emotional intelligence, and motivation [7, 8, 9]. There is increasing evidence from the field of neuroscience suggesting that meditation practice can enhance employees’ job performance via its influence on the autonomic nervous and endocrine systems. Therefore, meditation practice may be an effective intervention strategy to improve job performance in the business world.

Despite the theoretical plausibility linking meditation practice to job performance, previous studies examining meditation in the workplace have focused on stress reduction and depression [10, 11]. Few studies have examined the direct relationship between meditation practice and job performance. Furthermore, the prevalence of meditation practice and the characteristics of those who practice meditation are still unknown. Therefore, in the present study, we sought to (1) estimate the prevalence of meditation practice, (2) identify the characteristics of those who practice meditation, and (3) examine the association between meditation practice and job performance among Japanese businesspersons via cross-sectional data.

### Study Design

Study 1 was a cross-sectional study of adults in Japan that estimated the prevalence of meditation practice and the background characteristics of those that practice.

Study 2 was a cross-sectional study of Japanese business persons that examined the associations between the frequency of meditation practice and scores on the Utrecht Work Engagement Scale (UWES-9), the World Health Organization Health and Work Performance Questionnaire (HPQ), and a job satisfaction scale; demographic characteristics and other behavioral risk factors were controlled for in these analyses.

## Method

### Participants

#### Study 1

A population-based, cross-sectional survey targeting Japanese business people was conducted through the Internet in August 2014. We contacted registered participants of MARSH Co., Ltd., an Internet research company in Japan. MARSH Co. has access to more than 720,000 people to recruit as participants for survey studies, including the present study. Specifically, they invited 98,907 registrants, 44,872 of who accessed the survey. Registrants who indicated that they were students, unemployed, clergy, or instructors were excluded from the analysis. Students and unemployed were excluded since the aim was to examine meditation in businesspersons. Clergy and instructors were excluded because they may have been meditation experts. The final sample resulted in 30,665 participants (Table 1). The participants received online shopping points as an incentive.

**Table 1:** Demographic characteristics of the sample by meditation practice (*N* = 30,665)

		Meditation		Non-meditation group		
		Group (*n* = 1,208)		(*n* = 29,457)		
		*n*	%	*n*	%	*p*-value
All		1,208	3.9	29,457	96.1	
Age	<20	0	0.0^b^	13	0.0^b^	< 0.001^a^
	20–29	142	11.8	1,919	6.5	
	30–39	407	33.7	6,571	22.3	
	40–49	391	32.4	11,294	38.3	
	50–59	268	22.2	9,660	32.8	
Gender	Male	489	40.5	20,037	68	< 0.001^a^
	Female	719	59.5	9,420	32	
Education	Elementary school, junior high school	10	0.8^b^	335	1.1^b^	< 0.001^a^
	High school, vocational school	281	23.3	9,806	33.3	
	Junior college, technical college	172	14.2	3,274	11.1	
	University	665	55	14,365	48.8	
	Graduate school	80	6.6^b^	1,677	5.7^b^	
Household Income (Japanese Yen)	Less than 2 million	73	6.0^b^	1,428	4.8^b^	< 0.001^a^
	2–5 million	349	28.9^b^	9,062	30.8^b^	
	5–7 million	248	20.5^b^	6,436	21.8^b^	
	7–10 million	219	18.1^b^	5,883	20.0^b^	
	10–15 million	151	12.5	2,753	9.3	
	More than 15 million	71	5.9	811	2.8	
	Unknown	97	8	3,084	10.5	

^a^ Chi-square test.

^b^ Statistically significant standardized residual (*p* < 0.05).

#### Study 2

A population-based, cross-sectional survey targeting Japanese businesspeople was conducted through the Internet in August 2014. Registered participants of MARSH Co., Ltd. who were between the ages of 20 and 65 were recruited. Once again, students, unemployed individuals, clergy, and instructors were excluded as participants. We aimed to collect data from 1,600 participants; specifically, we sought to collect data from a total of 600 individuals who meditated (300 men, 300 women) and 1,000 individuals who did not meditate (500 men and 500 women). However, we could not collect data from an adequate number of individuals who meditated. Therefore, in order to collect enough data from individuals who meditated we increased the distribution of the questionnaires. As a result, questionnaires were distributed to 2,404 adults in Japan, and 1,603 participated. Of those who participated, we excluded the data from individuals who did not report household income (*n* = 122) and data with some error (*n* = 11). This resulted in a final sample size of 1,470 persons (Table 2). The participants received online shopping points as an incentive.

**Table 2:** Comparison of the background characteristics for the meditation group (n = 418) and non-meditation group (n = 1,052).

		Meditation Group (n = 418)				Non-meditation Group (n = 1,052)				
		Mean	(*SD*)	*n*	(%)	Mean	(*SD*)	*n*	(%)	*p*-value
Age	20–29			40	(9.6)^c^			82	(7.8)^c^	0.09^a^
	30–39			135	(32.3)			285	(27.1)	
	40–49			149	(35.6)^c^			390	(37.1)^c^	
	50–59			94	(22.5)			295	(28)	
Gender	Male			209	(50.0)^c^			531	(50.5)^c^	0.74^a^
	Female			209	(50.0)^c^			521	(49.5)^c^	
Education	Elementary school, junior high school			1	(0.2)^c^			13	(1.2)^c^	<0.001^a^
	High school, vocational school			93	(22.2)			317	(30.1)	
	Junior college, technical college			50	(12.0)^c^			141	(13.4)^c^	
	University			242	(57.9)			529	(50.3)	
	Graduate school			52	(7.7)			32	(4.9)	
Household Income (Japanese yen)	Less than 2 million			32	(7.7)^c^			67	(6.4)^c^	0.02^a^
	2–5 million			141	(33.7)^c^			381	(36.2)^c^	
	5–7 million			79	(18.9)^c^			238	(22.6)^c^	
	7–10 million			82	(19.6)^c^			228	(21.7)^c^	
	10–15 million			58	(13.9)			102	(9.7)	
	More than 15 million			26	(6.2)			36	(3.4)	
Marital Status	Unmarried			185	(44.3)			396	(37.6)	<0.001^a^
	Married			183	(43.8)			550	(52.3)	
	Common-law marriage			7	(1.7)			4	(0.4)	
	Divorced			39	(9.3)^c^			94	(8.9)^c^	
	Bereavement			4	(1.0)^c^			8	(0.8)^c^	
Stress responses (subscale of BJSQ)		19.0	(6.61)			18.3	(6.56)			0.05^b^
Body mass index (kg/height (m)^2^)		21.4	(3.13)			22.1	(3.82)			<0.001^b^
Physical activity at work		1.7	(0.8)			1.6	(0.84)			0.46^b^
Physical activity at leisure time		2.7	(1.09)			2.2	(1.1)			0.00^b^
Sleep hours		6.1	(1.11)			6.1	(1.07)			0.86^b^
Sleep sufficiency		2.2	(0.77)			2.2	(0.76)			0.75^b^
Work engagement (UWES-9)		3.0	(1.21)			2.7	(1.15)			<0.001^b^
Job performance (HPQ)		7.0	(1.94)			6.6	(1.98)			<0.001^b^
Job Satisfaction		27.6	(7.13)			26.5	(7.26)			0.01^b^

^a^ Chi-square test.

^b^ Student's t-test.

^c^ Statistically significant standardized residual (*p* < 0.05).

#### Ethics

Based on ethical guidelines for epidemiological research provided by the Japanese Ministry of Health, Labour and Welfare [12], an ethical review was not required for this study for the following reasons: 1) based on the Act on the Protection of Personal Information in Japan, the authors provided the participants with the privacy policy and the participants agreed to the use of their answers for analysis under anonymity; and 2) the analysis involved the use of unlinkable, anonymous, and publicly available data provided by Campus for H., Inc. (see S1 Dataset and S2 Dataset). Therefore, this study does not require an ethical review according to the Japanese guidelines.

### Measurements

#### Stress responses

Stress responses were assessed using the subscales of the Brief Job Stress Questionnaire (BJSQ) [13]. This questionnaire was developed with support from the Japanese Ministry of Health, Labour and Welfare and is used in Japan on a regular basis. Stress responses were measured by nine items, reflecting fatigue (e.g., “I am completely tired.”), anxiety (e.g., “I feel ill at ease.”), and depression (e.g., “I feel depressed.”). The BJSQ items were scored on a 4-point Likert scale, ranging from “1 = never” to “4 = always.” Each item was scored so that high scores indicated a higher level of stress response. It is believed that it is difficult to discriminate between different types of psychological distress in the workplace [14, 15]. Therefore, we used an aggregate of perceived stress responses as the independent variable; this was created by summing all nine items. In this sample, the Cronbach’s alpha coefficient was 0.94.

#### Subjective job performance

Subjective job performance was assessed using a single item from the HPQ [16]. Participants were asked to rate their overall job performance during the past four weeks on a 0–10 self-anchored scale, with 0 defined as the “worst possible work performance a person could have on this job” and 10 defined as “top work performance on the job.” We used this single-item, self-report global scale because (1) it has been argued that a global index of overall job performance (single item measure) is an inclusive and valid measure of job performance [16], (2) data on the objective performance of participants is difficult to obtain, and (3) alternative self-report measures of job performance focus on single occupations and include questions tailored to the unique demands of those occupations.

#### Work engagement

The nine-item Japanese version of the UWES-9 [17, 18] was used to assess work engagement. The UWES-9 was developed by Schaufeli et al. [17] and includes subscales measuring vigor (three items), dedication (three items), and absorption (three items). Items are rated on a seven-point response scale ranging from “0 = never” to “6 = always (everyday).” A total score on the UWES-9 (ranging from 0–6) was calculated by averaging item scores across the three subscales. The UWES-9 was translated into Japanese and has acceptable internal consistency reliability as well as factor and construct validities [18]. In this sample, the Cronbach’s alpha coefficient was 0.96.

#### Job satisfaction

Job satisfaction was assessed via nine items from the Japanese government cabinet office’s Kokumin Seikatsu Senkoudo Chousa (National Life Preference Survey) [19]. We assessed satisfaction with following questions: 1) relationships in the workplace, 2) employment conditions, 3) rewards, 4) increases in earnings, 5) employment stability, 6) sense of satisfaction at work, 7) difficulty in taking a vacation, 8) work life balance, and 9) diversity in the type of employment. These items were scored on a 5-point Likert scale, ranging from “1 = dissatisfied” to “5 = satisfied.” Each item was scored such that high scores indicated a higher level of satisfaction. An aggregate level of job satisfaction was used as the independent variable and was created by summing the nine items. In this sample, the Cronbach’s alpha coefficient was 0.89.

#### Sleep quality

Sleep quality was assessed via two items that were developed by Liu et al. [20]. The first item was “Do you feel rested after a night’s sleep?” This item was rated on a 4-point scale that ranged from “1 = very insufficient” to “4 = very sufficient.” The second question asked participants to report how many hours they sleep in an average night. These items were developed with support from the Japanese Ministry of Health, Labour and Welfare and are used in Japan on a regular basis [21].

#### Physical activity

Physical activity was assessed using two items created and validated by Johansson and Westerterp [22]. These items measure physical activity at work and at leisure time. The first item was: “Describe your physical activity while at work (even work at home, sick leave at home, and studying),” and was rated from “1 = very light” to “4 = heavy.” The second question asked participants to rate the following question: “Describe your physical activity at leisure time. If the activities vary between summer and winter, try to give an average estimate.” This question was rated on a scale ranging from “1 = very light” to “5 = very active.”

#### Demographic characteristics

In terms of demographic characteristics, we asked about participants’ gender, age (years), marital status (unmarried/married/common-law marriage/divorce/widowed), household income (JPY; less than 2 million/2–5 million/5–7 million/7–10 million/10–15 million/more than 15 million/unknown), and education (elementary school; junior high school/high school; vocational school/junior college; technical college/university/graduate school).

### Statistical analysis

In Study 1, descriptive analyses were conducted to estimate the prevalence of meditation practice and the characteristics of individuals who meditate. In Study 2, hierarchical multiple regression analyses were conducted and examined the effect of meditation on the three outcome variables (work engagement, subjective job performance, and job satisfaction). The independent variables were entered into the equation as follows: frequency of meditation practice was entered on the first step; demographic characteristics (gender, age, marital status [organized as a dummy variable: never married/divorced/widowed = 1, married/common-law marriage = 2], household income, education) were entered as controls on the second step; and behavioral risk factors (body mass index, physical activity at work, physical activity at leisure time, number of hours of sleep, sleep sufficiency) were entered on the third step to compare their effect with that of meditation practice. The level of significance was set to 0.05 (two-tailed). The statistical analyses were conducted using SPSS 22 for Windows.

## Results

### Study 1

The descriptive data are presented in Table 1. The prevalence of meditation practice was 3.9% for the current sample. Participants were divided into two groups and were compared on the dependent variables; the two groups were individuals who meditated (*n* = 1,208) and individuals who did not meditate (*n* = 29,457). As shown in Table 1, those who practiced meditation were younger than were those who did not practice meditation. In addition, individuals who practiced meditation reported significantly higher education levels and higher household incomes. The two groups also differed in gender; specifically, the proportion of female participants was higher in the group that practiced meditation (59.5%) than in the group that did not practice meditation (32.0%).

### Study 2

A comparison of the background characteristics between individuals who meditated (*n* = 418) and those who did not meditate (*n* = 1,052) is displayed in Table 2. When compared with the non-meditation group, the meditation group reported significantly higher education levels, household incomes, stress responses, physical activity at leisure time, work engagement, subjective job performance, subjective job satisfaction, and significantly lower body mass index (BMI). The two groups also differed in marital status. The proportion of married participants was lower in the meditation group (43.8%) than in the non-meditation group (52.3%).

#### Work engagement

The results of the hierarchical regression analysis for work engagement are presented in Table 3. As expected, meditation practice significantly accounted for 1.3% of the variance in the prediction of work engagement (*p* < .001). Demographics were added in the second step and accounted for a significant increase in 4.4% of the variance explained in the prediction of work engagement (*p* < .001). Behavioral risk factors were added in the third step and significantly accounted for 1.8% of the variance in the prediction of work engagement (*p* < .001; Table 3).

**Table 3:** Associations between meditation practice, demographic characteristics, behavioral risk factors, and work engagement: Results from the hierarchical regression analysis (740 males and 730 females).

Variables		Pearson *R*		Standardized coefficient (*β*)					
		Crude		Step 1		Step 2		Step 3	
1	Meditation practice								
	Frequency of practice^a^	0.112	***	0.112	***	0.106	***	0.086	***
2	Demographic characteristics								
	Gender^b^	0.004				0.032		0.044	
	Age (years)	0.086	***			0.070	**	0.075	**
	Education^c^	0.114	***			0.078	**	0.084	**
	Household income^d^	0.163	***			0.119	***	0.115	***
	Marital status^e^	0.081	**			0.024		0.020	
	Stress responses	-0.103	***			-0.081	**	-0.068	*
3	Behavioral risk factors								
	Body mass index	-0.026						-0.009	
	Physical activity at work^f^	0.046						0.076	**
	Physical activity at leisure time^g^	0.158	***					0.101	***
	Sleep hours	0.025						0.025	
	Sleep sufficiency^h^	0.061	*					0.011	
	*R* ^2^			0.013		0.056		0.074	
	Adjusted *R* ^2^			0.012		0.052		0.067	
	Δ *R* ^2^			0.013	***	0.044	***	0.018	***

*: *P* < 0.05,

**: *P* < 0.01,

***: *P* < 0.001.

^a^ Not currently practicing = 1, Less than one day in two weeks = 2, Once a week or more = 3, Daily or more = 4.

^b^ Male = 1, Female = 2.

^c^ Elementary school/junior high school = 1, High school/vocational school = 2, Junior college/technical college = 3, University = 4, Graduate school = 5.

^d^ Less than 2 million = 1, 2–5 million = 2, 5–7 million = 3, 7–10 million = 4, 10–15 million = 5, More than 15 million = 6 (JPY).

^e^ Never married/Divorced/Widowed = 1, Married/Common-law marriage = 2.

^f^ Very light = 1, Light = 2, Moderate = 3, Heavy = 4.

^g^ Very light = 1, Light = 2, Moderate = 3, Active = 4, Very active = 5.

^h^ Very insufficient = 1, Insufficient = 2, Sufficient = 3, Very sufficient = 4.

When the individual variables were examined, meditation practice (*β* = .112, *p* < .001) was significantly predictive of work engagement, and remained significant after controlling for demographic characteristics and behavioral risk factors (*β* = .086, *p* < .001). Furthermore, physical activity at leisure time was the strongest predictor of work engagement (*β* = .101, *p* < .001). Physical activity at work (*β* = .076, *p* = .004) was also positively predictive of work engagement.

#### Subjective job performance

The results of the hierarchical regression analyses for HPQ score are presented in Table 4. As expected, meditation practice significantly accounted for 1.3% of the variance in the prediction of HPQ (*p* < .001). In the second step, demographics accounted for a significant increase in the variance explained (18.9%) in the prediction of HPQ (*p* < .001). Behavioral risk factors were added in the third step and accounted for a significant 1.3% of the variance in the prediction of HPQ (*p* < .001; Table 4).

**Table 4:** Associations between meditation practice, demographic characteristics, behavioral risk factors, and the health and work performance questionnaire: Results from the hierarchical regression analysis (740 males and 730 females).

Variables		Pearson *R*		Standardized coefficient (*β*)					
		Crude		Step 1		Step 2		Step 3	
1	Meditation practice								
	Frequency of practice^a^	0.116	***	0.116	***	0.120	***	0.108	***
2	Demographic characteristics								
	Gender^b^	0.115	***			0.139	***	0.163	***
	Age (years)	0.091	***			0.057	*	0.057	*
	Education^c^	0.080	**			0.039		0.048	*
	Household income^d^	0.194	***			0.125	***	0.123	***
	Marital status^e^	0.112	***			0.059	*	0.057	*
	Stress responses	-0.378	***			-0.352	***	-0.331	***
3	Behavioral risk factors								
	Body mass index	-0.035						0.030	
	Physical activity at work^f^	-0.007						0.069	**
	Physical activity at leisure time^g^	0.155	***					0.071	**
	Sleep hours	0.059	*					-0.014	
	Sleep sufficiency^h^	0.212	***					0.051	
	*R* ^2^			0.013		0.202		0.215	
	Adjusted *R* ^2^			0.013		0.198		0.208	
	Δ *R* ^2^			0.013	***	0.189	***	0.013	***

*: *P* < 0.05,

**: *P* < 0.01,

***: *P* < 0.001.

^a^ Not currently practicing = 1, Less than one day in two weeks = 2, Once a week or more = 3, Daily or more = 4.

^b^ Male = 1, Female = 2.

^c^ Elementary school/junior high school = 1, High school/vocational school = 2, Junior college/technical college = 3, University = 4, Graduate school = 5.

^d^ Less than 2 million = 1, 2–5 million = 2, 5–7 million = 3, 7–10 million = 4, 10–15 million = 5, More than 15 million = 6 (JPY).

^e^ Never married/Divorced/Widowed = 1, Married/Common-law marriage = 2.

^f^ Very light = 1, Light = 2, Moderate = 3, Heavy = 4.

^g^ Very light = 1, Light = 2, Moderate = 3, Active = 4, Very active = 5.

^h^ Very insufficient = 1, Insufficient = 2, Sufficient = 3, Very sufficient = 4.

When the individual variables in these models were examined, meditation practice (*β* = .116, *p*< .001) was significantly predictive of HPQ scores, and these results held after controlling for demographic characteristics and behavioral risk factors (*β* = .108, *p* < .001). Furthermore, when examining the behavioral risk factors and meditation practice as predictors, meditation practice was the strongest predictor of HPQ (*β* = .108, *p* < .001); thus, it appears that frequent meditation practice was associated with increased performance. In addition, physical activity at work (*β* = 0.069, *p* = .005) and physical activity at leisure time (*β* = 0.071, p = .004) were also positively predictive of HPQ.

#### Job satisfaction

The results of the hierarchical regression analyses predicting job satisfaction are presented in Table 5. As expected, meditation practice accounted for a significant 0.6% of the variance in the prediction of job satisfaction (*p* = .002). Demographic variables, added in the second step, accounted for a significant increase in the amount of variance explained (13.8%) in the prediction of job satisfaction (*p* < .001). Behavioral risk factors were added in the third step and accounted for a significant 0.9% of the variance in the prediction of job satisfaction (*p* = .007).

**Table 5:** Associations between meditation practice, demographic characteristics, behavioral risk factors, and job satisfaction: Results from the hierarchical regression analysis (740 males and 730 females).

Variables		Pearson R		Standardized coefficient (*β*)					
		Crude		Step 1		Step 2		Step 3	
1	Meditation practice								
	Frequency of practice^a^	0.079	**	0.079	**	0.072	**	0.065	**
2	Demographic characteristics								
	Gender^b^	0.044				0.059	*	0.059	*
	Age (years)	-0.001				-0.052	*	-0.050	
	Education^c^	0.125	***			0.065	**	0.062	*
	Household income^d^	0.258	***			0.205	***	0.194	***
	Marital status^e^	0.125	***			0.062	*	0.067	*
	Stress responses	-0.272	***			-0.248	***	-0.200	***
3	Behavioral risk factors								
	Body mass index	-0.021						0.026	
	Physical activity at work^f^	-0.097	***					-0.038	
	Physical activity at leisure time^g^	0.117	***					0.050	*
	Sleep hours	0.068	**					0.006	
	Sleep sufficiency^h^	0.195	***					0.083	**
	*R* ^2^			0.006		0.145		0.154	
	Adjusted *R* ^2^			0.006		0.140		0.147	
	Δ *R* ^2^			0.006	**	0.138	***	0.009	**

*: *P* < 0.05,

**: *P* < 0.01,

***: *P* < 0.001.

^a^ Not currently practicing = 1, Less than one day in two weeks = 2, Once a week or more = 3, Daily or more = 4.

^b^ Male = 1, Female = 2.

^c^ Elementary school/junior high school = 1, High school/vocational school = 2, Junior college/technical college = 3, University = 4, Graduate school = 5.

^d^ Less than 2 million = 1, 2–5 million = 2, 5–7 million = 3, 7–10 million = 4, 10–15 million = 5, More than 15 million = 6 (JPY).

^e^ Never married/Divorced/Widowed = 1, Married/Common-law marriage = 2.

^f^ Very light = 1, Light = 2, Moderate = 3, Heavy = 4.

^g^ Very light = 1, Light = 2, Moderate = 3, Active = 4, Very active = 5.

^h^ Very insufficient = 1, Insufficient = 2, Sufficient = 3, Very sufficient = 4.

When the individual variables in these models were examined, meditation practice (*β* = 0.079, *p* = .002) was significantly predictive of job satisfaction scores, and this result was consistent even after controlling for demographic characteristics and behavioral risk factors (*β* = 0.065, *p* = .009). Comparing behavioral risk factors and meditation practice, sleep sufficiency was the strongest predictor of job satisfaction (*β* = .083, *p* = .007), thereby suggesting that those who have better sleep are more likely to be satisfied with their jobs.

## Discussion

It has been argued that meditation practice has the potential to enhance individual job performance. The present study was conducted for three purposes: (1) to estimate the prevalence of meditation practice, (2) to identify the characteristics of those who practice meditation, and (3) to examine the association between meditation practice and job performance among Japanese business people. To our knowledge, this is the first study to investigate the direct relations between meditation practice and job performance via several types of variables. Moreover, no studies to date have examined these associations among business people with cross-sectional data after controlling for demographic and behavioral risk factors.

First, the results of the present study indicated that there was an association between meditation practice and the outcome variables, including subjective job performance, work engagement, and job satisfaction. Importantly, these associations were significant even after controlling for covariates. These results are consistent with previous studies, suggesting that meditation practice is positively associated with these variables [23, 24, 25]. Our study is novel in that our results indicated that meditation practice predicted job performance when demographic and behavioral risk factors were entered as controls. It is possible that the effects of meditation on job performance reflect improved cognitive and non-cognitive functioning that result from the biological changes associated with meditation. Indeed, neuroscience research has indicated that the mental processes used during meditation practice affect the brain, autonomic nervous system, and endocrine system [26]. These biological changes may result in improved cognitive and non-cognitive functioning in individuals who practice meditation; this, in turn, results in better job performance.

Second, the results of this study did not reveal any associations between BMI and any of the outcome variables. Previous studies have reported that obesity causes multiple health problems that can negatively affect job performance [27, 28, 29]. The fact that our data did not demonstrate this association may be because of the difference of BMI distribution in Japan versus Western countries. In Japan, the mean BMI of the population is 23.71 (SD 3.37) kg/m^2^ in 20- to 69-year-olds and the percentage of individuals with a BMI ≥ 30 is no more than 2–3% in the population. [30]. In this study, the mean BMI of the meditation group was 21.4 (SD 3.13) kg/m^2^ and that of the non-meditation group was 22.1 (3.82) kg/m^2^. Therefore, the BMI distribution in the current sample was quite small, and there were relatively few obese individuals.

However, physical activity at work and physical activity at leisure time were significant predictors of work engagement and subjective job performance. It may be that physical activity is linked to job performance due to (1) enhanced self-efficacy, (2) reduced stress, and (3) improved physical functioning [31, 32]. In the present study, other potential confounding factors (e.g., alcohol intake, sleep deprivation, television consumption, excessive eating) were not examined. Therefore, future studies should consider including these factors.

Third, the data presented in Table 1 indicate that there is an association between age and meditation practice. Specifically, the ratio of individuals in the younger age group (20–29 years old) who practiced meditation (11.8%) was higher than that of those who did not practice (6.5%). However, only 3.9% of the total sample indicated that they practiced meditation (see Table 1). This may be attributed to Rogers’ theory of diffusion of innovations; this theory assumes that early adopters are key for spreading new behavior. Specifically, early adopters are willing to take risks, have a higher social status, have financial liquidity, are social, and have close contact with scientific sources and interaction with other innovators. Therefore, their risk tolerance allows them to adopt technologies that may ultimately fail, and their financial resources help absorb these failures [33].

This may be true in our study. When examining the background characteristics of the meditation group (Table 2), the education and household income of the meditation group were significantly higher than were those of the non-meditation group. In addition, there was a higher ratio of individuals who practiced mediation among individuals who had completed a high level of education, especially among those who had completed university. Furthermore, there were more individuals who meditated than those who did not meditate among the participants who reported high incomes. Accordingly, education and household income appear to have an impact on meditation practice. This finding may be attributed to the theory of “inverse equity” [34]. This theory states that new behavior interventions start in higher socioeconomic status (SES) groups and later affect lower SES groups. Thus, our data suggest that new practices, such as meditation, may diffuse from individuals who are high in SES to individuals who are low in SES. Therefore, it appears that high SES is a common characteristic of individuals who meditate.

On the contrary, when individuals are older and report lower levels of education and household income, they are less likely to practice meditation. It is possible that meditation will increase in high SES groups but will not increase in low SES groups. Therefore, it seems important for researchers investigate ways to diffuse this practice among older age and lower SES groups.

Finally, although the association between meditation practice and job-related outcomes was statistically significant after controlling for demographic characteristics and behavioral risk factors, its association was weak (R^2^ = 0.074 for work engagement, R^2^ = 0.215 for subjective job performance, and R^2^ = 0.154 for job satisfaction.) There are several possible reasons for the weak relation. First, our measures of meditation practice remained crude. For example, we did not measure the duration of mediation practice. As such, it is possible that a relatively large percentage of the subjects were novices in meditation practice and, therefore, did not show improved job-related outcomes. However, a previous study indicated that a short duration of meditation practice is also beneficial [35]. Second, this may be attributed to reverse causation. Business people who associated themselves with poor job-related outcomes may have started meditating. Third, it is possible that meditation practice only slightly enhances job-related outcomes, and does not have a large impact on job-related outcomes.

While this study provided new evidence about individuals who practice mediation and the association between meditation and job performance, there were several limitations. First, the data was cross-sectional in nature; therefore, causality cannot be inferred. Future studies should use longitudinal designs to examine the causal relationship between meditation practice and job performance. Second, the use of the Internet to administer surveys may have excluded individuals who have low IT literacy. Third, there may be other variables that could be controlled for in the analyses; however, the major confounding variables were included in the analyses (e.g., gender, age, marital status, household income, and behavioral risk factors), thereby reducing the likelihood of bias in our results. Fourth, the generalizability of the results is limited given that the data were drawn exclusively from Japanese participants. Future studies should consider examining the relations between meditation practice and job performance in different cultures.

## Conclusion

Despite the limitations of the present study, the findings presented herein provide evidence for the implications of meditation practice and its association with job performance among Japanese business people. In addition, the study provides information about the prevalence of meditation in this population. Our findings indicate that meditation practice may have positive effects on enhancing multiple dimensions of job performance, including work engagement, subjective job performance, and job satisfaction; these associations are independent of an individual’s demographic characteristics and behavioral risk factors. Diffusing meditation practice among low SES population may prevent inequality due to differential job-performance.

## Supporting Information

S1 Dataset Anonymized Dataset Used for Study 1 (n-30,665). (XLSX)

S2 Dataset Anonymized Dataset Used for Study 2 (n = 1,470). (XLSX)

S1 Table Pearson's correlation coefficients among the study variables (740 males and 730 females). (XLSX)
