# Correction: The Association between Meditation Practice and Job Performance: A Cross-Sectional Study

**DOI:** 10.1371/journal.pone.0145140

**Published:** 2015-12-16

**Authors:** Koichiro Shiba, Masahiro Nishimoto, Minami Sugimoto, Yoshiki Ishikawa

There are errors in the author affiliations. The affiliations should appear as shown here:

Koichiro Shiba^1^, Masahiro Nishimoto^1^, Minami Sugimoto^1^, Yoshiki Ishikawa^1^

**1** Campus for H Inc., Tokyo, Japan
